# T_H_17 cells…Sorting the good out from the bad

**DOI:** 10.1038/s41392-020-00316-2

**Published:** 2020-09-19

**Authors:** Junwei Li, Le Jiang, Yongxiang Yi, Pradeep Kumar Sacitharan

**Affiliations:** 1 College of Veterinary Medicine, Qingdao Agricultural University, 266109 Qingdao, China; 2 Department of General Surgery, The Second Hospital of Nanjing, The Affiliated Hospital of Nanjing University of Chinese Medicine, #1 Zhongfu Road, Nanjing, Jiangsu Province China; 3 The Institute of Ageing and Chronic Disease, University of Liverpool, Liverpool, L7 8TX UK; 4 Xi’an Jiaotong-Liverpool University, Department of Biological Sciences, #111 Ren’ai Road, Suzhou Industrial Park, 215123 Suzhou, Jiangsu Province P. R. China

**Keywords:** Adaptive immunity, Neurodevelopmental disorders, Immunotherapy

A recent article by Wu et al.^1^ demonstrated how targeting inflammatory T_H_17 cells can be achieved by blocking glycolysis pathway genes. These findings could lead to potential drugs to selective target “bad” inflammatory T_H_17 cells, while not disturbing “good” homeostatic T_H_17 cells in patients with autoimmune diseases.^1^

Animal models and human studies have showed a key role for T_H_17 cells in the immune system’s defense against bacteria and fungi, as well as the development of autoimmune diseases, mediated by the secretion of IL-17.^2^ In addition, antigen-presenting cells, such as dendritic cells during the immune response, secrete IL-23, which in turn activates T_H_17 cells.^2^ The essential role of homeostatic T_H_17 cells in the immune response can be exacerbated into autoimmunity whereby T_H_17 cells turn pathogenic and drive tissue damage and pathogenesis in diseases, such as psoriasis, multiple sclerosis, and rheumatoid arthritis.^3,4^ Targeting pathogenic T_H_17 cells can treat autoimmune diseases, but existing approaches also inhibit homeostatic T_H_17 cells, thereby increasing the risk of infection.^5^ Hence, finding the differences between these two types of T_H_17 cells is essential to understand how to target pathogenic T_H_17 cells and not to disturb homeostatic T_H_17 cells. The authors wanted to decipher the difference between the cellular metabolism of homeostatic and pathogenic T_H_17 cells.

**Fig. 1:**
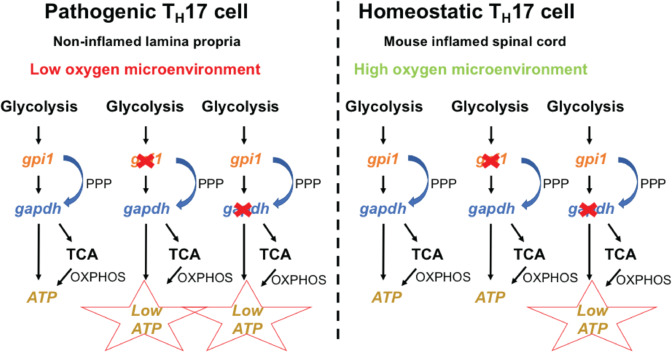
The glycolysis gene *Gpi1* is required for homeostatic T_H_17 cells in normal tissue whereby high oxygen levels allow pentose phosphate pathway (PPP) and OXPHOS to compensate for the loss of *Gpi1*. In hypoxic inflamed tissue, the loss of OXPHOS occurs, thus making *Gpi1* essential for the survival of pathogenic T_H_17 cells. Hence, metabolic redundancy varies according to the microenvironment, and *Gpi1* may be a therapeutic target in certain settings

The authors at first used an established mouse model of multiple sclerosis (Fig. 1), called experimental autoimmune encephalomyelitis (EAE), alongside mouse-knockout mosaic experiments to selectively differentiate pathogenic compared to homeostatic cells. Analysis of these elegant experiments revealed that the pathogenic cells were mostly T_H_17 cells. Furthermore, gene expression studies showed that pathogenic T_H_17 cells had a higher expression of genes associated with glycolysis compared to homeostatic T_H_17 cells. The authors went on to use sophisticated CRISPR-knockout mice studies and bone marrow chimeric experiments to tease out which genes in the glycolysis pathway were important to pathogenic T_H_17 cells. The team found that the gene Glucose Phosphate Isomerase 1 (*Gpi1*) is selectively required by pathogenic but not homeostatic T_H_17 cells. Mice containing cells deficient in *Gpi1* were unable to induce EAE.

At this point, the data showed that *Gpi1* was dispensable in the homeostatic model while other glycolysis genes are not. Wu et al. hypothesized that the pentose phosphate pathway (PPP) might maintain some glycolytic activity in the *Gpi1* KO cells and thus compensate for *Gpi1* deficiency. Data from both in vivo and in vitro experiments showed a significant increase in PPP activity in the *Gpi1* KO T_H_17 cells compared to controls, indicating that *Gpi1* deficiency maintains active glycolytic flux through PPP. The authors went on to knock out both *Gpi1* and *G6pdx*, which catalyzes the initial oxidative step in the PPP, to further test whether PPP activity compensates for *Gpi1* deficiency in vivo. The *Gpi1/G6pdx* double KO reduced cell number by about 75%, demonstrating that in vivo PPP activity maintains viability of homeostatic T_H_17 cells lacking *Gpi1*.

Next, the authors wanted to elucidate the impact of *Gpi1* deficiency on aerobic glycolysis activity. A reduced lactate production alongside a higher ATP-linked respiration rate was observed in *Gpi1* KO cells. These two results alongside additional in vivo validation pointed to the fact that mitochondrial respiration through pyruvate oxidation compensates for *Gpi1* deficiency in homeostatic T_H_17 cell differentiation. At this stage, the authors knew that the PPP and mitochondrial respiration were compensating for *Gpi1* deletion, but this suggested a partial metabolic redundancy for *Gpi1* via another compensatory mechanism. The authors performed kinetic testing alongside glucose labeling experiments, and showed that the loss of *Gpi1* led to a reduction of glucose uptake and abundance of pyruvate and lactate. Altogether, these data suggested that the reduced amount of glucose metabolized in *Gpi1* KO cells via PPP could support the production of glycolytic intermediates and maintain pyruvate oxidation. In addition, *Gpi1* KO cells increased their mitochondrial respiration to compensate for the loss of glycolytic flux.

For the final important question in this paper, the authors wanted to understand why pathogenic T_H_17 cells are particularly sensitive to *Gpi1* deficiency. They hypothesized that inflamed tissue that is hypoxic may lead to impaired mitochondrial respiration, resulting in an inability to generate ATP to compensate for energy loss due to *Gpi1* deficiency in these tissues. The authors cultured T_H_17 cells in hypoxic and normoxic conditions to show that *Gpi1* KO cells had decreased lactate production in both normoxic and hypoxic conditions. However, *Gpi1* KO cells only demonstrated reduced intracellular ATP in hypoxic conditions. Furthermore, in vivo analysis of inflamed tissue in an EAE model displayed decreased oxygen availability that was regulated by the oxygen sensor *Hif1a* in pathogenic T_H_17 cells.

Overall, Wu et al. revealed that T_H_17 cells increase glycolysis activity to adapt to the hypoxic environment in EAE. In this setting, reduced mitochondrial respiration cannot compensate for the loss of glycolytic ATP production upon *Gpi1* inactivation, leading to energy crisis and cell elimination. These results also provide the first proof that *Gpi1* inhibition may be a therapeutic option in disease states that display hypoxic microenvironments. However, we cannot exclude the possibility that other environmental factors, not only oxygen levels, may also play a role in these disease settings. Clinical trials and possible ex vivo experiments using disease tissue or cultured cells are required to validate these results further. Nonetheless, this paper opens a larger possibility of specifically inhibiting pathogenic cells by metabolic targeting of selective redundant cellular components, which opens up a myriad of therapeutic opportunities in different disease microenvironments.
